# Animal Models of *Pneumococcal pneumonia*

**DOI:** 10.3390/ijms20174220

**Published:** 2019-08-28

**Authors:** Noemi Borsa, Marta Di Pasquale, Marcos I. Restrepo

**Affiliations:** 1 Department of Pathophysiology and Transplantation, University of Milan, Internal Medicine Department, Respiratory unit and Adult Cystic Fibrosis Center, Fondazione IRCCS Ca’ Granda Ospedale Maggiore Policlinico, 20122 Milan, Italy; 2 Division of Pulmonary Diseases and Critical Care Medicine, The University of Texas Health Science Center at San Antonio, San Antonio, TX 78229, USA; 3 South Texas Veterans Health Care System; San Antonio, TX 78229, USA

**Keywords:** pneumonia, *S. pneumoniae*, animal model, antibiotic resistance, vaccine

## Abstract

*Streptococcus pneumoniae* remains the most common bacterial pathogen causing lower respiratory tract infections and is a leading cause of morbidity and mortality worldwide, especially in children and the elderly. Another important aspect related to pneumococcal infections is the persistent rate of penicillin and macrolide resistance. Therefore, animal models have been developed to better understand the pathogenesis of pneumococcal disease and test new therapeutic agents and vaccines. This narrative review will focus on the characteristics of the different animal pneumococcal pneumonia models. The assessment of the different animal models will include considerations regarding pneumococcal strains, microbiology properties, procedures used for bacterial inoculation, pathogenesis, clinical characteristics, diagnosis, treatment, and preventive approaches.

## 1. Introduction

Lower respiratory tract infections, as defined in the Global Burden of Diseases, Injuries, and Risk Factors Study (GBD) 2016, include pneumonia and bronchiolitis [1]. *Streptococcus pneumoniae* (known as pneumococcus) remains as the most common bacterial pathogen causing community-acquired lower respiratory tract infections and the leading cause of morbidity and mortality worldwide, contributing to more deaths than all other etiologies combined in 2016 (1.2 million deaths) [1,2,3,4]. The age groups more vulnerable and with the highest rate of deaths are children younger than five years and adults older than 70 years [1]. However, the death rate among children younger than five years decreased by 57% since 2000, and the incidence of pneumonia decreased by 21% over the same time period [1]. In contrast, the rate of deaths among adults aged 70 years and older increased from 746,700 in 2000 to 1,080,958 in 2016, despite the constant incidence rate of pneumonia [1]. This main difference is driven by 51% increased number of adults older than 70 years globally. In 2017, the World Health Organization (WHO) included *S. pneumoniae* as one of the 12 priority pathogens [5]. 

Pneumococcal pneumonia is characterized by an acute clinical presentation on a patient complaining of fever, chills, cough with productive sputum production, dyspnea, and pleuritic chest pain. The disease can progress to produce mental status changes, shock, and respiratory failure. In a physical exam, the most common findings include a febrile patient with signs consistent of a consolidation with asymmetric chest expansion, decreased breath sounds, crackles, bronchophony, echophony, and dullness to percussion. The most common laboratory findings include leukocytosis, or leukopenia if severe renal dysfunction, and decreased oxygenation. Imaging studies including chest radiograph, chest ultrasound, or chest tomography are confirmatory of the presence of pulmonary infiltrations that could be manifested as air bronchogram, areas of consolidation, and, on occasion, pleural effusion (Table 1).

Another important aspect related to pneumococcal infections includes the persistent rates of penicillin and macrolide resistance that represent a challenge for patients with this condition [6,7,8]. *S. pneumoniae* colonizes the nasopharynx starting early in life, for which reason it is difficult to determine the pathogenesis and the moment pneumococcus becomes invasive and causes serious diseases such as pneumonia [9,10]. Therefore, animal models were historically developed to better understand the mechanisms of disease, to test new diagnostic and treatment alternatives including those against highly resistant pneumococcus, and to test vaccines to prevent invasive disease [9,10]. This narrative review will focus on the characteristics of the different animal pneumococcal pneumonia models. The assessment of the different animal models will include considerations regarding the pneumococcal strains, microbiology properties, mode of bacterial inoculation, pathogenesis, clinical characteristics, diagnosis, treatment, and preventive approaches.

Since it is beyond the scope of the present review, viral (typically Influenza)-pneumococcal coinfection models will not be discussed here. 

## 2. Search Strategy and Selection Criteria

We searched Medline for any paper published in English language to March 1, 2019. We used the search terms “pneumonia” or “pneumococcal pneumonia” or “*Streptococcus pneumoniae*,” in combination with the terms “mouse model,” “rat model,” “rabbit model,” “swine model,” and “monkey model” and with the terms “pathogenesis,” “treatment,” “prevention,” “sepsis,” and their variations. We restricted the search strategy to pneumococcal pneumonia and sepsis models. We also searched the reference lists of articles identified by this search strategy. Review articles and book chapters are cited to provide readers with more details and more references.

## 3. Mouse Models of Pneumococcal pneumonia

The mouse is the most frequently encountered model of pneumococcal pneumonia. Among its diverse uses in literature, the most remarkable are the assessment of antibiotic efficacy, pharmacokinetics, disease pathogenesis, virulence factors, and vaccine testing analysis. 

Table 2 summarized purposes of mice models. 

### 3.1. Mouse and Pneumococcal Strains

In both humans and mice, pneumococcal infection is the result of a complex interplay between bacterial and host factors, which strongly influences disease severity and localization.

From the microorganism’s side, the bacterial capsule, which determines pneumococcal serotype, acts as a major virulence factor by protecting the microorganism from phagocytosis. Different capsular polysaccharides (and serotypes) are associated with different degrees of antiphagocytic activity and invasiveness [11,12]. Invasive and noninvasive strains can exist within the same serotype, due to variable expression of other virulence factors such as other cell wall components, pneumolysin [13,14,15,16], or other enzymes [17].

In humans, the pneumococcal serotype prevalence differs over time, by age group and by geographic area, and is influenced by vaccine use. Infection with serotypes 1, 2, and 3 typically leads to lobar pneumonia [18]. Serotype 1 in particular carries a high risk of complications, such as empyema [19]. The serotypes that are more likely to be found in patients with invasive disease (meningitis and bacteremia) are 4, 5, 6B, 7F, 19F, and 23F [20,21]. *S. pneumoniae* is also an important colonizer of the upper respiratory tract of children under five years of age, with serotypes 3, 6A, 6B, 15, and 19F being the most frequently detected in healthy carriers globally [22]. Conversely, serotypes 1, 4, and 5 rarely colonize the nasopharynx. In countries where pneumococcal vaccines are used, the prevalence of the aforementioned serotypes has dropped. However, despite a very significant decrease in the global incidence of invasive pneumococcal disease, a relative increase in colonization and infection with serotypes not covered by vaccine has been reported (an effect called “serotype replacement”) [23].

As for mice, *S. pneumoniae* is not part of the nasopharyngeal microbial community under normal circumstances. A study by Thevaranjan et al. published in 2016 found *Firmicutes*, *Proteobacteria*, *Bacteroidetes*, and *Actinobacteria* to be the most abundant phyla found in the upper respiratory tract of aging mice [24]. For this reason, colonization and infection need to be induced through experimental procedures. Some pneumococcal serotypes feature different virulence as compared to the human host. Generally, capsular serotypes 2–5 and 6B are highly virulent [12]. In contrast, types 1, 7F, 9, 14, 19F, and 23F tend to be less virulent [25,26,27]. Intranasal (IN) challenge with serotypes 4 (especially strain TIGR4) and 6B causes pneumonia in >90% animals and is usually associated with high levels of bacteremia [28,29,30,31]. Serotype 19F generally carries low virulence [12] in immunocompetent mice; for this reason, to induce infection with this serotype, most of the studies have traditionally used immunodeficient animals [32]. In addition, the number of bacteria inoculated and the method of inoculation also play huge roles in virulence of any particular strain. Study of the literature on the topic also showed a very high variability between lab to lab. 

On the host’s side, genetic background influences susceptibility to infection. Different mouse strains respond variably to pneumococcal challenge in terms of disease timing, severity, and outcome. Mouse strains can be classified into outbred and inbred. 

Most outbred strains were obtained from a single Swiss colony and currently include—amongst the most common—strains such as Caesarian Derived-1 (CD1), Institute of Cancer Research (ICR), Naval Medical Research Institute (NMRI), and Mike Flack-1 (MF1) [21]. These strains have higher genetic variability that allows to recreate a population’s different responses to infection [33]. The outbred Swiss strains have also been widely utilized to build a neutropenic model and to allow evaluation of infection with less virulent pneumococcal serotypes and strains, such as 9, 14, 19F, and 23F. Induction of neutropenia is achieved though IP injections with an immunosuppressant drug such as cyclophosphamide, with administration schemes that tend to show slight variations from author to author in terms of timing and dose. Generally, cyclophosphamide is started three or four days prior to infection. Following administration, neutrophil counts usually start to drop, reach nadir values on the day of infection and return to normal between 3–5 days after [34,35]. Under these conditions, neutropenic mice usually become bacteremic within 4 h [26,36] and develop acute bacteremic pneumonia and die within 2–4 days from infection, if untreated [35,36,37,38]. 

Given their highly selected genome, inbred strains such as C57BL/6J, CBA, and BALB/cJ have a more uniform response to infection. An example of this can be found in the N substrain of the CBA strain (CBA/N), which carries a X-linked inability to produce normal antibody responses to some types of antigens, including pneumococcal capsular polysaccharides and other thymus-independent antigens. This confers susceptibility to pneumococcal infections [39]. 

Another host factor involved in susceptibility to infection is age. Typically, mice are used as pneumococcal pneumonia models at a young adult age, which ranges between 6–14 weeks. Studies that specifically investigate the effect of aging on susceptibility to pneumococcal disease [31,40,41] compare aged mice (19–26 months old) with young adults. When correlating the entire lifespan, one human year is almost equivalent to nine mice days. However, this correlation varies according to the animal’s phase of life. For example, during the developmental period (from birth to weaning, which occurs at four months of age), one human year is equivalent to six mice days. Several authors have shown that some of the mechanisms underlying the variable response to pneumococcal infection in different age groups are due to modifications in inflammatory responses, such as delayed activation of inflammasome [42], delayed production of interleukin (IL)-1 and interferon (IFN) γ [43,44], increased production of chemokines [44], and Toll-like receptor dysfunction [29].

### 3.2. Routes of Infection

Several routes can be used to achieve lung infection—intratracheal (IT), aerosol, intravenous (IV), intranasal (IN), direct intrabronchial (IBr), and intraperitoneal (IP).

The IN and the IT techniques require the mouse to be anesthetized. Anesthesia is performed mainly via aerosol; other methods used are the IP and the intramuscular (IM) routes. For aerosol anesthesia, the animals are placed in a chamber where the anesthetic (usually isofluorane, halothane, or methoxyflurane) is vaporized. It has been suggested [45] that the anesthetic choice can influence the rate of invasive disease after IN challenge: inhaled halothane and methoxyflurane have been associated with greater number of bacteria in the lung and higher bacteremia at 24 h post-infection, as compared with IP pentobarbital injection. The most common medications used for IP anesthesia are ketamine and xylazine, which can also be administered by the IM route. Only rarely is anesthesia delivered through the IV route [46].

In the IT challenge, the pathogen is directly delivered into the animal’s respiratory tree. This can be obtained by using two main different techniques: the “oro-tracheal”/“peroral” and the “tracheal puncture” technique, respectively. In order to obtain infection via the oro-tracheal route, the first step is to suspend the animal vertically by its upper incisors [26,47,48,49,50]. The tongue is then displaced anteriorly with a retractor and the microorganism is instilled through the upper airways while the animal is inhaling [16,47,51,52,53]. To facilitate distal alveolar migration of the bacteria by gravity [54], the mice are held in a vertical position until anesthesia has waned (approximately 30 min for pentobarbital [45] and 2 min for inhaled anesthetics [45,55]). This technique has been shown to reproducibly deliver more than 99% of the inoculum to the lungs [16]. The tracheal puncture technique, although more invasive than the oro-tracheal, avoids contamination of the inoculum with oral flora [56] and has an unexpected low perioperative mortality (around 4%) and allows recovery within a few hours [57]. While the mouse is kept in a supine position, the neck is transilluminated with a light source, and a vertical midline incision is performed in order to expose the trachea, which is cannulated with a 22–30 G needle held parallel to it [58,59,60,61,62,63]. The wound is then either closed with surgical glue or sutured [58,59,62,63]. Some authors expose the trachea in order to visualize the position of a needle inserted through the mouth, allowing accurate administration of the bacteria without the need to incise the airways [61]. 

The IN route can be used to establish both nasopharyngeal colonization and infection, mainly depending on the number of bacteria contained in the inoculums and the degree of sedation of the animal. The position of the animal during instillation has shown little impact on delivery of the inoculum [64]. In the IN model, the bacterial suspension is applied into the nostrils through a pipette tip. To achieve infection, this procedure is carried out while the animal is under deep anesthesia, which suppresses gag and cough reflexes and allows inhalation. To facilitate distal migration of the inoculum to the alveoli, anesthetized animals are usually kept in supine position with their heads elevated at a 45-degree angle until anesthesia has waned [45]. A few studies suggest that the degree of sedation can determine whether IN instillation can result in colonization or infection: light anesthesia may preserve airway protection and prevent leakage of the bacterial suspension from the upper airways into the bronchial tree [65,66,67]. 

In the aerosol technique, the bacterial suspension is placed in a Venturi-type nebulizer driven by 10 L/min of 5% CO_2_ in either air or oxygen. The nebulizer is connected to an exposure chamber through polyethylene tubing, with a similar efflux system containing a low-resistance microbial filter and connected to a biosafety hood [30,68]. The mice are placed in the exposure chamber for approximately 20 min [30]. The CO2 content in the aerosol promotes deep ventilation [30,68,69]. Some authors utilize chambers with individual compartments to prevent the animals from huddling and assure uniform exposure to the aerosol and decontaminate the mice coats with UV light for 5 min [70]. As for the IN route, this model can be used to establish both infection [70] and colonization [71] depending on virulence of the strain or susceptibility of mice. Of note, in 1996, Iizawa and colleagues [71] elaborated a model in which nasopharyngeal carriage was achieved through the aerosol route and pneumonia was subsequently induced by airway obstruction caused by IT instillation of 20 μL of 2% formalin.

As summarized by Hoover et al. in 2017 [72], another method described to induce lung infection is direct intrabronchial instillation. This technique was first used in rat models and later modified for mice. The animal is intubated orotracheally with a metal cannula that is advanced generally into the left main bronchus. The metal cannula is then used as a guide to advance polyethylene tubing, through which the inoculum is instilled (usually 20 µL).

The IV and IPe routes are mainly used to induce systemic pneumococcal infection that leads to bacteremia and then a possible secondary pneumonia [7]. For IV administration, the bacteria are usually suspended in normal saline and then injected through a 25G needle [45], usually through the tail vein [45,71] or—less commonly—the retro-orbital plexus [73].

Every infection model offers advantages and disadvantages. In general, the oro-tracheal, the direct intratracheal, and the direct intrabronchial models offer a more precise delivery of the inoculum directly into the airways. This makes them ideal for inducing infection with less virulent pneumococcal serotypes. We believe that the technique that features the best balance between advantages and disadvantages is the oro-tracheal: it is fast and minimally traumatic for the animal. The main downside of its lower invasiveness is however that the operator must be skilled at injecting the inoculum with the right timing while the animal is inhaling. On the other hand, the oro-tracheal is more invasive and requires the operator to have specific surgical skills but is independent of the animal’s respiratory cycle. The direct intrabronchial infection method does not require surgical skills but can result technically difficult give the small size of the animal. The main advantage of the IN and aerosol models is that they reproduce the natural history of pneumococcal infection by inducing upper airway colonization. However, they are not usually adequate to cause pneumonia with less virulent serotypes.

A technique similar to the one used for intrabronchial and intratracheal instillation also allows collection of bronchoalveolar lavage (BAL) to determine drug concentrations in the animal’s lung [13]. To collect BAL, a small volume (usually 1 mL) of sterile saline is instilled into the animal’s lungs and immediately removed. The sample is then centrifuged to remove cellular debris.

### 3.3. Particular Models of Pneumococcal Pneumonia in Mice

Some models have been created in order to specifically assess the impact of comorbid conditions on pneumonia development and disease progression. As mentioned earlier in the “Mouse and pneumococcal strains” section above, Swiss mice have been largely used in literature to build the neutropenic model. A study evaluating the efficacy of CpG-Oligodeoxynucleotide treatment in pneumococcal pneumonia secondary to trauma used a model of third-degree burn [70]. Other studies [74] assessed pneumococcal pneumonia pathogenesis in emphysematous patients by inducing emphysema through IT instillation of porcine pancreatic elastase. Chronic alcohol diets and IPe injections of 20% alcohol in saline were used to build a model of acute-on-chronic alcohol intoxication and study its effect on granulocyte response during pneumococcal pneumonia [75], showing reduced response due to impaired granulopoietic progenitor cell proliferation.

Another model, which will not be discussed here as it is beyond the scope of this review, is the co-infection model that uses a viral infection (typically influenza virus) prior or concomitant with pneumococcal inoculation, a situation commonly encountered in clinical practice.

## 4. Rat Models

Animal models of pneumonia using rats are less common than mouse models. The main advantages in comparison to mouse are the bigger size and therefore the amenability to perform some procedures and the possibility of using a larger bacterial burden. The major aims of studies on rat models of pneumococcal pneumonia consist in collecting data on pathogenesis, survival, bacterial counts in lung and blood, pathological and histological characteristics, drug efficacy, and vaccine evaluation both in adults and children (Table 3).

The most common rat strains used for experimental purposes are Wistar, Sprague Dawley, and CD rats [76]. The Wistar rat is a hybrid albino strain and the first one to serve as an animal model, in general. The Sprague Dawley rat is another hybrid outbred albino rat, derived from the Wistar rat. It is usually chosen for its calmness and easiness to handle and also has a high reproduction rate and low incidence of spontaneous tumor. The CD rat has a long history of use in toxicology research. These animals were obtained from Sprague-Dawley rats in the 1950s and rederived to improve the health status of the animals.

Usual routes of inoculum in rat models are IT, intrabronchial (IB), or intrapulmonary (IPu). Inoculation can be both surgical and non-surgical [76]. The surgical technique consists in exposing the apical lobe and delivering bacteria into several apical lobe bronchi through the bronchial wall into the lumen [77]. The nonsurgical method is based on IB instillation of bacteria resuspended in cooled melted agar via intubation, inducing a robust lung infection [78]. Another group used nonsurgical transthoracic inoculation of pneumococci entrapped in cooled agar particles into the mid-right lungs [79]. 

Among the studies focusing on pathogenesis, we observed that rat models are suitable to address lobar pneumococcal pneumonia, in particular on the role of impaired phagocytosis and on the detrimental effect of pneumolysin, which enhance pneumococcal virulence [78,79,80]. For example, it was demonstrated a role of pneumolysin in decreasing complement components and reducing serum opsonic activity [81]. Another study on pneumococcal sepsis from pulmonary origin evaluating sodium bisulfide (NaHS) as inflammatory drug, registered alterations of physiological variables 4 h after the IT inoculum [82]. 

Rats models are also generally used to measure the inoculum size. The inoculum size needed to cause pneumococcal infections after IT inoculation ranged from 5.7 to 6.2 log10 CFU/mL [82,83]. Supernatants obtained from bronchoalveolar lavage are usually used to measure ante e post-mortem inflammatory levels of studied biomarkers and to detect their concentration and activation in the lungs during pneumococcal pneumonia [82,84].

Rat models of experimental pneumonia are also used to assess the virulence of different pneumococcal serotypes, especially in children [79]. Moreover, penicillin-resistant pneumococcal strains and other resistant phenotypes have been established in the existing rat models in order to evaluate the efficacy of new antibiotics [78,83]. 

Rat models are also considered suitable to evaluate pneumonia in immunocompromised hosts, especially in asplenic, neutropenic, cirrhotic, or alcoholic subjects [76]. The majority of studies focused on models related to alcoholism and cirrhosis, which are two important pneumococcal risk factors in humans and carry a high mortality rate (up to 40%), respectively [85,86]. The chronic ethanol intoxication model was created by a continuous ethanol administration [87]. Cirrhosis is induced by administration of a hepatotoxin that causes cirrhosis and ascites [88]. Both models showed an increased susceptibility to pneumococcal pneumonia similar to what is seen in human subjects. 

In our opinion, the major and more important application of the rat model is the assessment of the efficacy, pharmacodynamic/pharmacokinteic of new antibiotics for susceptible or pneumococcal resistant strains (e.g., penicillin-resistant pneumococcal strains) [72,76,83,85,89,90].

Rat models are also useful to focus on pneumococcal sepsis, especially on conditions related to immunodepression, such as post-splenectomy. This model has been used to assess survival, host-pathogen responses (e.g., complement function), and bacterial pathogenicity (e.g., bacterial load in the blood) [76,88,91,92]. We think that rat pneumococcal sepsis models are attractive models for testing new drugs that have the potential to improve outcomes in sepsis. Previous experimental models challenged rats with sodium bisulfide (NaHS) that resulted in a reduction of sepsis-related lung and kidney injury, while the host defenses remained intact [82]. Therapies adjunctive to antibiotics such as systemic corticosteroids and gammaglobulins have also been tested in rat models, particularly in splenectomized rats with great success [93,94]. The administration of anticoagulants is another novel therapeutic use to defeat pulmonary coagulopathy during pneumonia in rat models [84]. 

In addition to therapeutic options, rat pneumococcal pneumonia models are also generally used to assess antipneumococcal vaccination efficacy and safety [95].

Finally, rat models are, in our opinion, also suitable to evaluate the pathogenesis of pneumococcal infections complications. For example, a rat model was used to detect infected pleural effusion by dosing complement activation product levels in pleural fluid of animals [83].

## 5. Rabbit Models

Pneumococcal pneumonia rabbit models are suitable for researchers to study pathogenesis, survival, disease progression (i.e., measurements of white blood cells levels in lungs and blood and histological changes in lungs), and pharmacokinetic and pharmacodynamic characteristics of novel therapeutic and immunization agents [76] (Table 3).

Generally, rabbit models are used to study pneumococcal pneumonia and sepsis pathogenesis and test drug efficacy. However, in our opinion there are some limitations: fewer models are available in respect to mice and rats, mainly because of a lack of well-equipped animal houses, of expert handlers of the animal, and a scarcity of literature on the care of experimental utilization of rabbits [76,96]. New Zealand white strains of rabbits are commonly being used for research activities. This strain is less aggressive in nature and has less health problems as compared with other breeds [96]. Less frequently used are chinchilla rabbits, usually for pneumoccal sepsis models. This group of rabbits has been bred for a coat that resembles that of chinchillas, and they are also accustomed to being handled by humans [97]. Another breed found as a pneumonia model is Dutch-belted rabbits, which, due to their smaller size, require approximately 40% less drug than studies conducted with New Zealand White rabbits [98].

Pulmonary infection in rabbit models is usually delivered IT, IB, or IP, respectively. The first described rabbit model to our knowledge was based on instillation of pathogens through a catheter into the trachea [99]. Subsequent models developed a method of IB instillation of bacteria [100,101].

As in all animal models, also in rabbit models of pneumococcal pneumonia establish inhaled or aspirated bacterial load threshold that determines the development of bacterial pneumonia is a standard part of model development and it is repeated every time a new strain is used in the model [102].

Pathogenesis is one of the major goals of rabbit models, according to our research. One original rabbit model was used to study the effect of pneumococcal cell surface components and to assess the role of the platelet-activating factor in the pathogenesis of pulmonary inflammation and pneumonia, respectively [99,103]. In addition, the rabbit model has been used to examine the role of host oligosaccharides in preventing pneumococcal colonization of the nasopharynx and the time to subsequent lung infection [104].

Rabbit models are also normally useful to assess different pneumococcal serotypes [18]. Penicillin-resistant pneumococcal strains and other resistant phenotypes have been established in rabbit models in order to evaluate efficacy of new antibiotics [18,101,105,106]. The Piroth model mimicked lethal pneumococcal lobar pneumonia with subsequent parenchymal consolidations and bacteremia [101]. The model is very advantageous to evaluate pharmacokinetic and efficacy of different drugs, which makes rabbit models unique tools for testing efficacy of compounds, even if they are not used so often for this purpose, especially respectful to mouse models [101,105,106,107]. The main focus is on molecules against resistant strains of pneumococci, especially against strains of *S. pneumoniae* with different resistance patterns to penicillin [107,108].

Finally, rabbit models are also suitable to study and evaluate pneumococcal sepsis. Pneumococci inoculated by IP administration allow researchers to assess for clinical parameters measured during the progression of the disease [109]. Different studies used the same model to assess pathogenesis of pneumococcal sepsis, metabolic alterations, especially in splenectomized hosts, and drug efficacy [108,109,110]. In addition, subsequent studies focused on the role of the spleen in clearing pneumococci during experimental sepsis created by intracardiac inoculation [111,112]. As for other animal models, IV sepsis rabbit models are also appropriate to evaluate pharmacokinetic properties of antibiotics (e.g., cephalosporins), often alongside efficacy studies to allow for PK/PD correlation [113].

## 6. Swine Models

Swine models (i.e., pigs) are used to replicate pneumonia infection due to its similarity with humans in terms of anatomy, genetics and physiology [114,115,116,117] (Table 3). Swine models allow various surgical and non-surgical procedures typically used in human medicine. Swine models offer a wide range of standardized methodologies due to their size compared to other small animal models [115]. In addition, lungs and upper respiratory tract of swine are similar to the human organs, except for the presence of a fourth lobe in the right lung [116,117].

Swine models can be a result of a naturally developed pneumonia due to a porcine pathogen (*Streptococcus suis)*, which cause a pneumonia similar to that occurring in humans. Alternately, other experimental surrogate infection models are the result of an infection created by a human pathogen given to a pig (eg. human pneumococcal serotypes 8 and 6b) [114].

According to us, swine models are best suited to evaluate ventilator-associated pneumonia. Unlike pneumonia acquired in the community, this requires the animals to be critically ill, placed in prone position (to prevent atelectasis) on invasive mechanical ventilation [118]. The use of prophylactic antibiotics and the clinical presentation usually leads to a lethal process in a short period of time (3–4 days), representing a hyperacute process [119]. Derived from these experiments, swine models allow researchers to obtain data for biochemical measures, blood gas analysis, physiological variables, ventilatory settings, pre- and post-mortem cultures, blood and bronchoalveolar lavage samples for inflammatory markers, antibiotic pharmacokinetics/pharmacodynamics, and pathology analyses [119,120,121,122,123,124]. This hyperacute model showed interesting translational results and it is useful for the study of the local and systemic responses of lung infection and for the determination of potential measures of prevention or therapeutic modulation [114]. The advantages of an animal model of VAP are based on the accurate control of significant variables such as the precise timing of the infectious challenge, the effect of antimicrobial therapy on cultures, and the possibility to perform accurate pharmacokinetic and pharmacodynamic studies on drugs [119]. Because of methodological limitations regarding tissue sampling in humans, an animal model is required in order to assess the quantitative deposition in the whole lung parenchym of intravenously administered and, especially, inhaled antimicobials [119]. In particular, cultures of tracheal secretions, bronchoalveolar lavage (BAL), protectedspecimen brushes (PSB), and direct lung aspirates were compared with cultures of lung homogenates and with histological findings [119].

To our knowledge, limited information is available regarding swine models of pneumococcal infection. A swine model of pneumococcal infection, used to assess pneumococcal invasive disease after *S. pneumoniae* serotype 8 IV administration, developed bacteremia with high pneumococcal bacterial burden within 48 h post inoculation [125]. The same group attempted to mimic oropharyngeal colonization, simulating a carrier state using a swine model in which pneumococcal serotype 6B was by IN inoculation. The study showed by polymerase chain reaction (PCR) that pneumococcus persisted in the oropharynx for at least seven consecutive days [126]. In addition, authors found that some animals developed bacteremia, suggesting that pneumococcus is able to become invasive and elude the host defense barriers. This pneumococcal swine model is a valuable model for studies on colonization, transmission and development of vaccines and directed therapies against pneumococcus. In addition, invasive bacteremic pneumococcal swine models allow researchers to assess pathogenesis (e.g., marked platelet activation and hyperreactivity reactions), subsequent vascular complications, and assess therapies to prevent these complications (i.e., by using antiplatelet inhibitors) [127].

Finally, we recommend swine models are also very useful for vaccine research, because vaccines can be administered by IM, subcutaneous, intradermal, oral, or IN routes. Collection of body secretions and samples is feasible with this model, making it attractive for immune response follow-up [114].

## 7. Non-Human Primates

In our opinion, research utilizing non-human primates (NHPs) for pneumococcal pneumonia is limited compared to other to other types of experimental models. The use of smaller animal models (e.g., mouse, rats, rabbits, etc.) has advantages in terms of feasibility due to cost, access, and availability. Nevertheless, NHPs are preferred models due to the similarities with humans, in terms of the ability to translate pathophysiological, diagnostic, therapeutic and preventive methods, and interventions, respectively (Table 3).

It is suggested that pneumococcus is not naturally carried by NPHs. Studies on infant rhesus macaques (*n* = 158) lack to show nasopharyngeal colonization. However, after experimental infant rhesus (*n* = 8) nasopharyngeal instillation of human *S. pneumoniae* strain (19F), 100% of the animals had evidence of colonization at two weeks and 60% at seven weeks post instillation, confirming that rhesus macaques may serve as a human-like carriage model [128].

There is an important interest in assessing whether NHP including rhesus monkeys could be suitable to assess pneumococcal components (e.g., IgA protease, capsular polysaccharide) and the relationship with pathogenesis (i.e., production of antibodies) during pneumococcal infection [129,130,131].

Another advantage of NPH models is that they develop symptoms consistent with lower respiratory tract infection similar to what is seen in humans. For instance, squirrel monkeys given IT 10^5^–6 × 10^8^ colony-forming units (CFU) of *S. pneumoniae* died four to six days later after developing severe illness characterized by fever, bacteremia, lethargy, anorexia, coughing, labored breathing, and bronchopneumonia [132]. Similar findings were seen in rhesus macaque models that develop similar respiratory symptoms, elevation of inflammatory biomarkes and pro-inflammatory cytokines in serum and BAL [133].

NHPs models are, in our opinion, also suitable for studies on inoculation ad pathogenesis. NHP studies on baboons (*n* = 15) confirmed that escalating doses of pneumococcal inoculation (serotype 19A-7) produce a host response ranging from spontaneous clearance of 10^6^ CFU to severe pneumonia 10^9^ CFU. Selected BAL fluid and plasma cytokine levels and RNA profiles were associated with radiological confirmed severe pneumonia [134]. A different group of investigators showed that, in a baboon model infected by IB inoculation with pneumococcus (serotype 4) 10^9^ CFU, the animals developed signs and symptoms of pneumonia four days post-infection. Clinical findings were similar to the one developed in humans, and include cough, tachypnea, dyspnea, tachycardia, and fever. All animals developed leukocytosis and bacteremia 24 h after infection. A severe inflammatory reaction was detected by elevation of serum cytokines, including Interleukin (IL) 1Ra, IL-6, and IL-8 after infection. Lung ultrasonography consistently identified pneumonia affected lobes correlated by pathological analyses. Lung pathology positively correlated with disease severity. Antimicrobial therapy rapidly reversed symptomology and reduced serum cytokines [135].

To our knowledge, the potential application of PK profile or use of BAL in NHP models has not been evaluated. However, in our opinion, NHPs could be perfectly used for that purpose, especially because of methodological limitations regarding tissue sampling in humans. Therefore, an animal model is required in order to assess the quantitative deposition in the whole lung parenchyma of intravenously administered and, especially, inhaled antimicrobials. In particular, cultures of tracheal secretions, bronchoalveolar lavage (BAL), protected specimen brushes (PSB), and direct lung aspirates were compared with cultures of lung homogenates and with histological findings.

It is not clear whether vaccination may fully protect NHPs to pneumococcal disease, particularly in chimpanzees [136,137]. Cardiac complications post-pneumococcal pneumonia have been reported in NHPs. In a severe pneumonia baboon model, investigators confirm that *S. pneumoniae* is capable of invading the myocardium and induce cardiac injury with necroptosis and apoptosis, followed by cardiac scarring after antibiotic therapy [138].

Additionally, NHPs models are appropriate to conduct interventional studies to assess the potential mechanisms by how therapies such as inhaled carbon monoxide may have a therapeutic potential for patients with acute respiratory distress syndrome (ARDS) [139,140,141]. Investigators showed evidence of ventilator-compatible carbon monoxide inhalational delivery system that may accelerate the resolution of ARDS in a clinically relevant NHP model mentioned above (i.e., baboon) [141].

## 8. Conclusions

This comprehensive narrative review attempted to describe the different characteristics of the most commonly used animal models in pneumococcal science. With the lenses of translational science this narrative review attempted to summarize relevant preclinical data on rodents, rabbits, swine, and non-human primates challenged with pneumococcal pneumonia infection. These models were created to test the impact of multiple interventions such as therapeutic and immunization agents. In addition, this review expanded the use of the different animal pneumococcal pneumonia models including relevant information about microbiological pneumococcal properties, mode of bacterial inoculation, pathogenesis, and the direct correlation with clinical disease as the one observed in humans. Further detailed in-depth reviews should focus on specific intervention targets in order to advance the science regarding pneumococcal disease and help improve outcomes of patients with pneumococcal pneumonia.

## Figures and Tables

**Table 1:** Pneumococcal pneumonia in humans.

**Clinical presentation**	fever chills cough productive sputum dyspnea pleuritic chest pain
**Complications**	mental status changes shock respiratory failure
**Physical exam**	signs of a consolidation with asymmetric chest expansion decreased breath sounds crackles bronchophony echophony dullness to percussion
**Laboratory findings**	leukocytosis or leukopenia renal dysfunction decreased oxygenation
**Imaging (CXR, CT, lung US)**	pulmonary infiltrations air bronchogram areas of consolidation occasion pleural effusion

Table legend. CXR: chest X-rays; CT: computerized tomography; US: ultrasounds.

**Table 2:** Summary of the specific aims of the mouse models cited in the present review.

Purpose of the Model	Specific Aim	References
TREATMENT	Antibiotic efficacy testing	[13,25,26,27,34,35,36,37,38,40,46,48,52,53,54]
PREVENTION	Vaccine efficacy testing	[23,32]
PATHOGENESIS	Bacterial capsule as virulence factor	[12,18,19,20]
	Virulence factors other than capsule	[11,16,17]
	Host defence mechanisms against pneumococcal infection	[29,30,31,33,39,41,42,43,44,49,50,55,56,57,58,60,61,65,66,73]
	Role of airway obstruction in pathogenesis	[71]
	Mechanisms of tissue damage in pneumococcal pneumonia	[29,47]

**Table 3:** Summary of the specific aims of the animal models cited in the present review.

Type of Model				
Purpose of Model	Rat	Rabbit	Pig	NHP
TREATMENT [References]	Efficacy of new antibiotics [72,76,83,85,89,90]	Evaluate pharmacokinetic and efficacy of different drugs [101,105,106,107,108]	Development of directed therapies against pneumococcus [125,126,127]	Test new drugs for ARDS [139,140,141]
	Test new drugs for sepsis [82,84,93,94]	Test new drugs in pneumococcal sepsis [113]	Therapies against inflammatory complications [125,126,127]	
PREVENTION [References]	Test vaccination efficacy and safety [95]		Development of vaccines [114,125,126,127]	Test vaccination efficacy and safety [136,137,138]
PATHOGENESIS [References]	Impaired phagocytosis [76,77,78,79,80]	Measure inoculum size [99,100,101,102]	Evaluate VAP [114,119,120,121,122,123,124]	Pneumococcal colonization [128]
	Effect of pneumolysin [77,78,79,80,81]	Effect of pneumoccoccal cell surface components [99,103]	Assess pneumococcal invasive disease [125,126,127]	Assess pneumococcal components [129,130,131]
	Measure inoculum size [82,83]	Role of the platelet-activating factor on lung inflammation [99,103]	Evaluate oropharyngeal colonization [125,126,127]	Measure inoculum size [134,135]
	Measure inflammatory levels in the lungs [82,84]	Role of host oligosaccharides [104]	Evaluate pneumococcal transmission [125,126,127]	Assess severity of illness [132,133]
	Assess virulence of different serotypes [78,79,83]	Assess different pneumococcal serotypes [18,101,105,106]	Evaluate platelet activation and hyperreactivity reactions [125,126,127]	
	Evaluate pneumonia in immunocompromised host [76,85,86,87,88]	Pneumococcal sepsis [108,109,110,111,112]		
	Pneumococcal sepsis [76,88,91,92]			
	Evaluate pneumococcal complications [83]			
